# Effect of inclusion rate of silage with or without alpha-amylase trait on finishing steer growth performance, carcass characteristics, and agronomic efficiency measures

**DOI:** 10.1093/tas/txaa056

**Published:** 2020-05-09

**Authors:** Warren C Rusche, Julie A Walker, Zachary K Smith

**Affiliations:** Department of Animal Science, South Dakota State University, Brookings, SD

**Keywords:** α-amylase, corn silage, feedlot, finishing cattle, integrated systems

## Abstract

One hundred ninety-two Continental × British steers [initial body weight (BW) = 420 kg (standard deviation = 24.7)] were used in a randomized complete block design finishing study to evaluate the effects of feeding two types of silage germplasm at two inclusion rates. A 2 × 2 factorial arrangement of treatments was used with either a conventional hybrid (Golden Harvest G07B39-311A, Syngenta Seeds LLC, Minnetonka, MN; CON) or a hybrid with increased expression of alpha-amylase (Syngenta Enogen Feed corn, Golden Harvest E107B3-3011A-EVT5, Syngenta Seeds, LLC; ENO) fed at either 12% (12SIL) or 24% (24SIL) of diet dry matter. Steers were blocked by source and location (source 1: first three pen replicates, *n* = 10 steers per pen with a fourth pen replicate of six steers per pen; source 2: one pen replicate, *n* = 12 steers per pen) and assigned randomly within block to treatments, resulting in five pens and 48 steers per treatment. Steers were harvested after 126 (12SIL) or 140 (24SIL) days on feed (DOF). There were no silage hybrid by inclusion rate interactions detected for live growth performance (*P ≥* 0.15). Silage hybrid did not affect average daily gain (ADG), gain-to-feed ratio (G:F), or final BW (FBW; *P* ≥ 0.35). Feeding 24% silage reduced ADG (*P* = 0.04) and increased G:F (*P* = 0.01) but increased FBW (*P* = 0.02) because of greater DOF compared with 12SIL. A hybrid by inclusion rate interaction was detected (*P* = 0.04) for calculated yield grade (YG) with steers fed 24SIL having increased YG within CON but not ENO. Hot carcass weight and rib fat were unaffected by silage hybrid (*P* ≥ 0.81) but were increased by feeding 24SIL (*P* = 0.03 and *P* = 0.02, respectively). Feeding increased amounts of silage increased beef produced per hectare (*P* = 0.05). Source of silage did not affect feedlot growth performance of cattle but, because of slight differences in estimated silage yield, conventional silage produced more kilograms of beef per hectare (*P* < 0.01). Feeding increased amounts of silage reduced G:F on both a live and carcass-adjusted basis but increased kilograms of beef produced per unit of land, which is paramount to cattle feeders who grow their own feedstuffs.

## INTRODUCTION

Corn silage is a cornerstone feed ingredient for beef production in the Midwest. It is a versatile source of readily digestible energy and neutral detergent fiber (NDF) and can be an effective option for marketing home-raised feedstuffs through cattle. The most effective use of corn silage is in growing cattle diets. In finishing diets, corn silage is typically limited to the minimum amount required for sufficient scratch factor to maintain ruminal health (Samuelson et al., 2016). However, farmer feeders may increase the utilization of silage for several reasons, including weather conditions, workload demands, or market signals. Increased inclusion rates of corn silage in finishing diets may be economically beneficial, depending upon the business and marketing strategies of the enterprise and the degree of integration between crops and livestock (Goodrich et al., 1974; DiCostanzo et al., 1997; Klopfenstein and Hilscher, 2018). Measuring the efficiency of beef production both on a unit of cropland and on a per animal basis is important in an integrated crop–livestock system.

Corn hybrid selection affects the amount of beef produced per hectare of cropland because of differences in both yield and digestibility. Recently, corn hybrids with an increased expression of an alpha-amylase enzyme have been marketed as a method to enhance starch digestion either when fed as grain or as corn silage. Others have noted that silages from these hybrids have increased feed efficiency in growing (Johnson et al., 2019) and finishing cattle diets (Baker et al., 2019). The objective of this study was to evaluate the effects of levels of corn silage inclusion [on a dry matter (DM) basis] with or without alpha-amylase on the growth performance and carcass characteristics of finishing yearling steers and to determine differences in efficiencies as measured on both a per animal and per unit of cropland basis. Our hypothesis was that feeding silage with alpha-amylase would increase gain-to-feed ratio (G:F) and that increasing DM inclusion of corn silage would result in lesser average daily gain (ADG) and G:F on a live and carcass-adjusted basis but increase beef produced per unit of cropland.

## MATERIALS AND METHODS

All procedures involving the use of animals in this experiment were approved by the South Dakota State University Institutional Animal Care and Use Committee (IACUC, approval number 19-008E). The experiment was conducted at the South Dakota State University Southeast Research Farm (SERF) located near Beresford, SD.

### Experimental Design and Treatments

A randomized complete block design was used to evaluate animal performance, carcass traits, and beef produced per hectare. Treatments were arranged in a 2 × 2 factorial with the factors of silage hybrid (conventional silage, CON, Golden Harvest G07B39-311A, Syngenta Seeds LLC, Minnetonka, MN) or (Syngenta Enogen Feed corn silage, ENO, Golden Harvest E107B3-3011A-EVT5, Syngenta Seeds, LLC) and corn silage inclusion at either 12% (12SIL) or 24% (24SIL) of diet DM (Table 1). The two corn hybrids were genetically similar except for the expression of the alpha-amylase trait. Both hybrids were planted on May 9, 2018, at a population of 74,132 plants per hectare. Plots received the same amounts of commercial fertilizer and identical herbicide treatments during the growing season. Silage harvest occurred on September 10, 2018 (CON) and September 11, 2018 (ENO). Silage was stored in oxygen impermeable bags using two bags for each hybrid.

**Table 1.** Actual diet formulations fed^*a*^

	Finisher (day 22 to harvest)			
Silage^*b*^	CON		ENO	
Inclusion^*c*^	12	24	12	24
Dry-rolled corn, %	65.1	52.9	65.0	52.7
MDGS, %^*d*^	19.3	19.7	19.3	19.6
Silage, %	11.5	23.3	11.6	23.5
Liquid supplement, %^*e*^	4.1	4.1	4.1	4.2
Nutrient composition^*f*^				
DM, %	65.8	59.0	67.1	60.3
CP, %	12.4	13.2	12.8	13.2
NDF, %	15.3	20.9	15.9	19.6
ADF, %	6.3	9.7	7.1	9.5
Ash, %	4.6	5.7	4.7	5.0
NE_m_, Mcal/ kg	2.11	2.04	2.10	2.04
NE_g_, Mcal/kg	1.43	1.37	1.43	1.37

^*a*^All values except DM on a DM basis.

^*b*^Silage hybrid: CON, conventionally available corn silage without α-amylase trait; ENO, silage from Syngenta Enogen Feed Corn.

^*c*^Dietary DM inclusion: 12, 12% inclusion of diet DM as corn silage; 24, 24% inclusion of diet DM as corn silage.

^*d*^MDGS, modified distillers grains plus solubles.

^*e*^Provided 30 g/ton of monensin, as well as vitamins and minerals to exceed requirements (NASEM, 2016).

^*f*^Tabular NE from Preston (2016) and actual nutrient compositions from monthly composite samples of the diets.

### Animals, Initial Processing, and Study Initiation

A total of 192 [initial body weight (BW) 420 kg (SD 24.7)] steers were used in this study. Steers were sourced from two different consignments at one South Dakota sale barn and delivered to the SERF. Source 1 steers (*n* = 144 steers; first three pen replicates, *n* = 10 steers per pen with a fourth pen replicate of six steers per pen) and source 2 steers (*n* = 48 steers; pen replicate 5; 12 steers per pen) were received on March 25, 2019. Cattle were processed on March 28, 2019, BW was collected, a unique identification tag was applied to each steer, and cattle were vaccinated against respiratory pathogens: infectious bovine rhinotracheitis (IBR), bovine viral diarrhea (BVD) types 1 and 2, parainfluenza-3 virus (PI3), and bovine respiratory syncytial virus (BRSV; Bovi-Shield Gold 5, Zoetis, Parsippany, NJ) and clostridial species (Ultrabac 7/Somubac, Zoetis). On April 2, 2019, steers were administered pour-on moxidectin (Cydectin, Bayer, Shawnee Mission, KS), administered a steroidal implant (200 mg trenbolone acetate and 28 mg estradiol benzoate; Synovex Plus, Zoetis), BW collected, and the study was initiated.

### Diet and Intake Management

Steers were fed once daily in the morning. Bunks were managed to be slick at 0800 h most mornings. Steers were stepped up to their final diet over a 21-d period with three step-up diets utilized. Feed intake and diet formulations were summarized at weekly intervals. Steers that died during the trial or that were removed from the study were assumed to have consumed feed equal to the pen mean dry matter intake (DMI) up to the point of removal or death. Three steers (two from the ENO-12SIL and one from the CON-12SIL treatments, respectively) died during the study from issues unrelated to dietary treatment; thus, all data are reported on a deads and removals excluded basis.

Ingredient samples were collected weekly and DM calculated after drying in a forced-air oven at 60 °C. Weekly DM values for each ingredient were used to calculate DMI and actual DM ingredient inclusions. Bunk samples were also collected weekly and stored in a freezer at −20º C until nutrient analyses were completed. After DM determination (method no. 935.29; AOAC, 2012), weekly samples from the final step for each treatment were composited into a monthly sample of the diets. The monthly composite samples of the finishing diets were analyzed for nutrient composition (N, method no. 968.06; AOAC, 2016; Rapid Max N Exceed; Elementar; Mt. Laurel, NJ; NDF and acid detergent fiber; Van Soest et al., 1991; and ash, method no. 942.05; AOAC, 2012).

### Cattle Management and Data Collection

Steer BW was recorded at the time of study initiation, on day 28 (pen BW), day 63, day 126, and day 140 (24SIL only) for the calculation of live growth performance. Body weights were measured before the morning feeding. A 3% pencil shrink was applied to final BW (FBW) and carcass-adjusted performance was calculated using hot carcass weight (HCW) adjusted to a common dressing percentage of 62.5%.

Cattle were shipped when they were visually appraised to have 1.27 cm of rib fat (RF). Cattle were shipped on two different dates: August 6, 2019 (12SIL) after 126 days on feed (DOF) and on August 20, 2019 (24SIL) after 140 DOF and harvested the following day at Tyson Fresh Meats in Dakota City, net energy (NE). Video image data was obtained from the plant for longissimus muscle (LM) area, RF, calculated USDA Yield Grade (YG), and USDA marbling scores. Dressing percentage was calculated as HCW/(FBW × 0.97). Carcass measurements were used to calculate empty body fat percentage (EBF; Guiroy et al., 2002), adjusted FBW at 28% EBF (AFBW), and proportion of closely trimmed boneless retail cuts from carcass round, loin, rib, and chuck (retail yield, RY; Murphey et al., 1960).

Performance-adjusted NE (paNE) was calculated from daily energy gain (EG; Mcal/d): EG = ADG^1.097^ × 0.0557W^0.75^, where W is the mean equivalent shrunk BW [shrunk BW × (478/AFBW), kg; (NRC, 1996)]. Maintenance energy required (EM; Mcal/d) was calculated by the following equation: EM = 0.0077BW^0.75^ (Lofgreen and Garrett, 1968), where BW is the mean shrunk BW from the trial. Using the estimates required for maintenance and gain, the paNE_m_ and paNE_g_ values (Owens and Hicks, 2019) of the diet were generated using the quadratic formula: $x=\;\frac{-b\pm \sqrt{b^{2}-4ac}}{2c}$, where *x* = NE_m_, Mcal/kg, *a* = −0.41EM, *b* = 0.877EM + 0.41DMI + EG, *c* = −0.877DMI, and NE_g_ was determined from: 0.877NE_m_ – 0.41 (Zinn and Shen, 1998; Zinn et al., 2008).

Beef production per hectare of cropland was calculated from DMI of corn silage and dry-rolled corn for each pen using the weekly diet compositions and DMI records. Actual corn silage yield observed at the Southeast Research Farm in September 2018 was 45.7 and 42.1 metric ton/ha for CON and ENO, respectively (P. Sexton, personal communication). Corn yield (kg/ha) was estimated using the formula: corn yield (kg/ha) = silage yield (as-is, metric ton/ha) × 224 (Lauer, 2006). Cropland required was the sum of kilograms consumed per yield for both corn and corn silage. Beef production (kg/ha) was then calculated as (carcass-adjusted FBW – initial BW)/ha.

### Statistical Analysis

Growth performance and carcass traits were analyzed as a randomized complete block design using the GLIMMIX procedure of SAS 9.4 (SAS Inst. Inc., Cary, NC) with pen as the experimental unit. The model included fixed effects of block, silage hybrid, inclusion rate, and the interaction of silage hybrid × inclusion rate. Least squares means were generated using the LSMEANS statement of SAS. Data means were separated and denoted to be different using the pairwise comparisons PDIFF and LINES option of SAS when a significant preliminary *F*-test was detected. An α of 0.05 or less determined significance, and tendencies are discussed from 0.05 to 0.10.

## RESULTS

Steer performance results are reported in Table 2. There were no silage × inclusion interactions (*P* ≥ 0.15) detected for any live or carcass-adjusted growth performance traits. Silage hybrid did not affect final live or carcass-adjusted BW, ADG, DMI, or G:F (*P* ≥ 0.35). Silage hybrid had no influence on paNE values (*P* ≥ 0.55) or observed/expected NE values (*P* ≥ 0.49).

**Table 2.** Animal growth performance, carcass characteristics, and efficiency measures^*a*^

	Silage type (S)		Inclusion rate (I)			*P*-values		
Item	CON	ENO	12%	24%	SEM^*b*^	S	I	S × I
Pens, *n*	10	10	10	10				
DOF, d	133	133	126	140				
Initial BW, kg	421	420	421	420	0.5	0.24	0.80	0.49
FBW, kg^*c*^	612	615	608	619	2.9	0.54	0.02	0.24
ADG, kg	1.44	1.47	1.49	1.42	0.021	0.35	0.04	0.17
DMI, kg	10.2	10.3	10.3	10.2	0.07	0.54	0.86	0.59
G:F	0.140	0.141	0.144	0.137	0.0016	0.45	0.01	0.15
Carcass basis^*d*^								
Final BW, kg	634	633	627	640	3.5	0.99	0.03	0.37
ADG, kg	1.60	1.61	1.64	1.56	0.025	0.80	0.06	0.27
G:F	0.156	0.155	0.159	0.152	0.0020	0.93	0.03	0.29
paNE, Mcal/kg^*e*,*f*^								
Maintenance	1.96	1.97	1.98	1.94	0.015	0.55	0.07	0.21
Gain	1.31	1.32	1.33	1.29	0.013	0.55	0.07	0.22
Tabular trial NE, Mcal/kg^*g*^								
Maintenance	2.05	2.05	2.08	2.02				
Gain	1.37	1.37	1.39	1.34				
Observed/expected NE^*h*^								
Maintenance	0.96	0.96	0.95	0.96	0.007	0.49	0.37	0.25
Gain	0.95	0.96	0.95	0.96	0.009	0.55	0.53	0.20

^*a*^Silage type: CON, conventionally available corn silage without α-amylase trait; ENO, silage from Syngenta Enogen Feed Corn. Dietary DM inclusion: 12, 12% inclusion of diet DM as corn silage; 24, 24% inclusion of diet DM as corn silage.

^*b*^Pooled SEM.

^*c*^FBW shrunk 3% to account for digestive tract fill.

^*d*^Calculated from HCW/0.625.

^*e*^pa = performance adjusted (Owens and Hicks, 2019).

^*f*^Calculated according to the equations provided by Zinn and Shen (1998) and Zinn et al. (2008).

^*g*^Tabular NE value weighted for each diet fed.

^*h*^paNE/tabular trial NE.

Final live and carcass-adjusted BW were 1.8 and 2.1% greater, respectively, for 24SIL compared with 12SIL (*P* ≤ 0.03). However, 24SIL steers required an additional 14 d on feed to reach a similar compositional endpoint as the 12SIL steers translating into a poorer (*P* = 0.04) live-basis ADG for the 24SIL steers. Daily DMI did not differ (*P* = 0.86) between 12SIL and 24SIL. Steers fed 12SIL had greater live (*P* = 0.01) and carcass-adjusted (*P* = 0.03) G:F compared with the 24SIL steers. Steers fed 24SIL tended to have lesser (*P* ≤ 0.07) paNE values compared with 12SIL steers and observed/expected NE values did not differ (*P* ≥ 0.37) between silage inclusion level.

**Figure 1.**
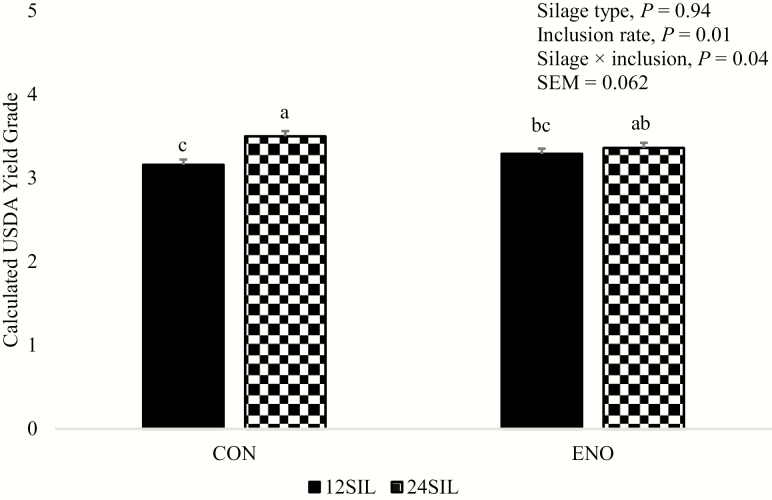
Interaction for calculated USDA Yield Grade between silage hybrid and inclusion rate. Corn silage was harvested from either a conventional hybrid (CON) or from a hybrid with increased α-amylase expression (Syngenta Enogen Feed Corn, Syngenta Seeds, LLC, Minnetonka, MN; ENO) fed at either 12% or 24% of diet DM (12SIL and 24SIL, respectively) in a randomized complete block design. For each of the four treatment combinations, there were 48 steers housed in five pen replicates. Means with different superscripts differ *P* < 0.05.

There were no silage × inclusion interactions detected for carcass traits except for YG (*P* = 0.04; Table 3). Silage hybrid did not affect dressing percentage, HCW, LM area, RF, marbling scores, kidney, pelvic, heart fat (KPH) percentage, estimated EBF, AFBW, YG, or RY (*P* ≥ 0.19). No differences were detected between 12SIL and 24SIL for dressing percentage, LM area, marbling score, KPH percentage, or FBW at 28% EBF (*P* ≥ 0.56). Silage hybrid interacted with inclusion rate (*P* = 0.04) with steers fed 24SIL having increased YG within the CON but not ENO treatments (Fig. 1). Feeding 24SIL did increase (*P* ≤ 0.03) HCW, RF, YG, and RY and tended (*P* = 0.06) to increase EBF compared with 12SIL.

**Table 3.** Carcass traits and beef production per ha of cropland^*a*^

	Silage type (S)		Inclusion rate (I)			*P*-values		
Item	CON	ENO	12%	24%	SEM^*b*^	S	I	S × I
Dressing percent, %^*c*^	64.67	64.38	64.47	64.50	0.191	0.30	0.70	0.83
HCW, kg	396	396	392	400	2.2	0.99	0.03	0.37
LM area, cm^*b*^	84.8	85.0	85.2	84.6	0.72	0.86	0.57	0.22
RF, cm	1.37	1.37	1.32	1.45	0.046	0.81	0.02	0.25
KPH, %	1.80	1.76	1.79	1.77	0.025	0.19	0.56	0.91
YG	3.33	3.33	3.23	3.43	0.044	0.94	0.01	0.04
RY, %^*d*^	49.82	49.86	50.04	49.63	0.098	0.80	0.01	0.06
Estimated EBF, %^*e*^	30.87	30.90	30.53	31.25	0.250	0.93	0.06	0.55
FBW at 28% EBF (AFBW), kg^*f*^	575	575	575	575	2.7	0.86	0.99	0.82
Marbling score^*g*^	532	510	519	522	12.7	0.25	0.85	0.39
Beef produced, kg/ha	2,121	1,974	2,008	2,087	25.7	<0.01	0.05	0.34

^*a*^Silage type: CON, conventionally available corn silage without α-amylase trait; ENO, silage from Syngenta Enogen Feed Corn. Dietary DM inclusion: 12, 12% inclusion of diet DM as corn silage; 24, 24% inclusion of diet DM as corn silage.

^*b*^Pooled SEM.

^*c*^HCW/FBW shrunk 3%.

^*d*^As a percentage of HCW.

^*e*^Estimated EBF and AFBW calculated according to the equations described by Guiroy et al. (2002).

^*f*^USDA Marbling Score 400 = Small^0^ = Low Choice; 500 = Modest^0^ = Average Choice.

There was no silage × inclusion rate interaction for beef production per hectare of cropland (*P =* 0.34; Table 3). Because as-is silage yield of CON was greater than ENO, conventional silage did produce (*P* < 0.01) more beef per hectare compared with ENO (2,121 vs. 1,974 ± 25.7 kg beef/ha, respectively). Feeding increased amounts of corn silage also resulted in greater production of beef per hectare compared with 12SIL (*P* = 0.05, 2,008 vs. 2,087 ± 25.7 kg beef/ha cropland, respectively).

## DISCUSSION

### Effect of Silage Type

The lack of response in this experiment to silage expressing the alpha-amylase trait contrasts with the positive effects observed when Enogen Feed corn silage was fed to growing steers (Johnson et al., 2019) or finishing yearling steers (Baker et al., 2019). In the growing cattle study by Johnson et al. (2019), feeding Enogen silage at 40% of diet DM without a corn processing coproduct increased ADG and tended to increase DMI, resulting in greater G:F. Feeding Enogen silage at 8% of diet DM combined with corn gluten feed in finishing diets reduced DMI with no effect on ADG, resulting in greater G:F compared with conventional silage (Baker et al., 2019). In contrast to the present experiment, neither of these studies utilized distillers grains as a source of supplemental protein. Both experiments also examined the effects of Enogen Feed corn as starch sources in the diets in a 2 × 2 factorial arrangement of treatments. Johnson et al. (2019) fed dry-rolled corn at 38.5% of diet DM, while Baker et al. (2019) used steam-flaked corn at 74.5% of diet DM. Including Enogen Feed corn as grain either resulted in no effect on cattle performance (Johnson et al., 2019) or reduced G:F (Baker et al., 2019) compared with conventional corn sources.

Similar inconsistencies have been observed in experiments utilizing corn with the alpha-amylase trait in finishing cattle, particularly when distillers grains are concurrently fed. Schoonmaker et al., (2014) compared corn grain expressing alpha-amylase at three different inclusion rates in diets containing wet distillers grains. They observed no differences in DMI, performance measures, or carcass characteristics. When Enogen Feed Corn was used as the sole source of starch in finishing cattle diets, positive responses for ADG and G:F were observed when corn gluten feed was included in the diet but in only one of two experiments that included distillers grains (Jolly-Breithaupt et al., 2019). Taken together, these results suggest that feeding Enogen Feed Corn does not elicit a consistent response when combined with distillers grains in finishing diets.

### Effect of Silage Inclusion Rate

The results of this experiment align well with previous work reporting that increased inclusion rates of corn silage in finishing cattle diets result in reduced ADG and G:F (Goodrich et al., 1974; Gill et al., 1976; DiCostanzo et al., 1997, 1998; Burken et al., 2017a, 2017b; Hilscher et al., 2019). This would be expected when corn silage replaces corn grain such as in this experiment because of the lesser NE_g_ for corn silage compared with corn grain (Owens et al., 2018). In this experiment, increasing silage inclusion rate by 12% decreased G:F by 4.4% compared with a 5.1% predicted decrease using regression values derived from studies with silage inclusion rates from 10% to 80% of diet DM (Goodrich et al., 1974).

Providing increased amounts of corn silage did not affect DMI in the current experiment. This result was surprising considering that increased NDF supply from roughage is associated with increased DMI (Galyean and Defoor, 2003). In the current experiment, NDF as percentage of DM was 4.5% greater in the 24SIL diet compared with 12 SIL. Assuming that this increase is a direct result of increased silage inclusion, predicted DMI should increase by approximately 0.6 kg/d for 24SIL based on the relationship between NDF supplied by forage and DMI from Galyean and Defoor (2003). In a review of feeding trials specifically evaluating corn silage inclusion, DMI was not markedly increased until inclusion rate exceeded 28% (Owens et al., 2018). Studies comparing silage inclusion rates at concentrations similar to those used in the present experiment noted increased DMI in some trials (Goodrich et al., 1974; Gill et al., 1976; DiCostanzo et al., 1997) but no differences in others (Brennan et al., 1987; DiCostanzo et al., 1998; Burken et al., 2017b; Hilscher et al., 2019). Thus, when corn silage is included at less than 30% DM, DMI is not consistently increased or decreased.

Another possible explanation for DMI results that are not consistent with previous literature is the warmer than normal temperatures experienced at Beresford, SD, during the last 80 d of the feeding period for the 24SIL treatment group. During that time period, the normal maximum heat index value was exceeded on 34 out of 80 d and the minimum observed heat index was greater than the normal minimum value on 44 out of 80 d (South Dakota Mesonet, 2020). Excessive heat loads are associated with decreases in DMI (Mader, 2003), which may have limited the willingness of steers on the 24SIL treatment to increase voluntary feed intake in the current experiment.

Other studies have reported reduced FBW and HCW as a result of increased corn silage inclusion where DOF were consistent between treatments (Burken et al., 2017a, 2017b; Hilscher et al., 2019). In this experiment, steers on the 24SIL treatment were fed an additional 14 d in an attempt to equalize final HCW with the expectation that increased silage would depress dressing percentage. Dressing percentage did not differ between 12SIL and 24SIL in the current experiment; consequently, steers fed an increased amount of corn silage had heavier BW at trial completion with greater HCW and increased RF compared with 12SIL. Dressing percentage has been shown to decrease in response to increased dietary corn silage in some studies (Brennan et al., 1987; Burken et al., 2017a; Hilscher et al., 2019) but remained unchanged in others (Gill et al., 1976) with Burken et al. (2017b) reporting reduced dressing percentage in one experiment but no differences in the second experiment. The additional DOF for the 24SIL treatment likely played a role in the increased RF and YG, reduced RY, and a tendency for increased EBF% observed in the current experiment. The additional HCW observed in the steers on the 24SIL treatment did not represent greater carcass weight associated with frame growth as evidenced by similar AFBW between the steers fed either 12SIL or 24SIL.

The interaction between silage type and inclusion rate for YG is not easily explained. Jolly-Breithaupt et al. (2019) observed increased RF and greater YG with Enogen Feed Corn fed with distillers grains. In the present experiment, the authors suspect that there is little biological significance to the differing YG responses observed.

### Beef Produced Per Unit of Cropland

Differences in beef produced per unit of cropland associated with feeding different silage types were entirely caused by different corn yields for the hybrids grown under these specific circumstances and not differences in growth performance or feed efficiency in the present study. Feeding increased amounts of silage resulted in greater amounts of beef produced per hectare. Johnson et al. (2016) observed that harvesting corn as silage, earlage, high-moisture, or dry corn did not affect gross return per hectare of cropland when utilized for finishing beef cattle. The optimum corn crop utilization strategy likely depends upon the interactions between corn price, business model (seasonal placement and marketing patterns vs. continuous occupancy), and the ability to capture manure value as part of an integrated crops-livestock system (Goodrich et al., 1974; DiCostanzo et al., 1997; Klopfenstein and Hilscher, 2018).

These data indicate that silage hybrids had no effect on animal growth performance or carcass traits but that choosing silage hybrids with greater yield does result in increased beef produced per hectare. Feeding increased amounts of silage resulted in reduced ADG and feed efficiency on an individual animal basis but increased HCW and beef produced per hectare compared with a reduced silage inclusion rate when fed to a numerically equal RF thickness. Cattle feeders that raise their own feed may be able to increase the amount of beef produced from a fixed land base by increasing the inclusion rate of corn silage in cattle finishing diets.
